# Proteomic characterisation of endoplasmic reticulum-derived protein bodies in tobacco leaves

**DOI:** 10.1186/1471-2229-12-36

**Published:** 2012-03-16

**Authors:** Minu Joseph, M Dolors Ludevid, Margarita Torrent, Valérie Rofidal, Marc Tauzin, Michel Rossignol, Jean-Benoit Peltier

**Affiliations:** 1 INRA, LPF UR1199, 2 Place Viala, 34060 Montpellier cedex, France; 2 Centre de Recerca en Agrigenòmica (CRAG)_CSIC-IRTA-UAB, Parc de Recerca UAB, Bellaterra (Cerdanyola del Vallés), 08193 Barcelona, Spain

## Abstract

**Background:**

The N-terminal proline-rich domain (Zera) of the maize storage protein γ-zein, is able to induce the formation of endoplasmic reticulum (ER)-derived protein bodies (PBs) when fused to proteins of interest. This encapsulation enables a recombinant fused protein to escape from degradation and facilitates its recovery from plant biomass by gradient purification. The aim of the present work was to evaluate if induced PBs encapsulate additional proteins jointly with the recombinant protein. The exhaustive analysis of protein composition of PBs is expected to facilitate a better understanding of PB formation and the optimization of recombinant protein purification approaches from these organelles.

**Results:**

We analysed the proteome of PBs induced in *Nicotiana benthamiana *leaves by transient transformation with Zera fused to a fluorescent marker protein (DsRed). Intact PBs with their surrounding ER-membrane were isolated on iodixanol based density gradients and their integrity verified by confocal and electron microscopy. SDS-PAGE analysis of isolated PBs showed that Zera-DsRed accounted for around 85% of PB proteins in term of abundance. Differential extraction of PBs was performed for in-depth analysis of their proteome and structure. Besides Zera-DsRed, 195 additional proteins were identified including a broad range of proteins resident or trafficking through the ER and recruited within the Zera-DsRed polymer.

**Conclusions:**

This study indicates that Zera-protein fusion is still the major protein component of the new formed organelle in tobacco leaves. The analysis also reveals the presence of an unexpected diversity of proteins in PBs derived from both the insoluble Zera-DsRed polymer formation, including ER-resident and secretory proteins, and a secretory stress response induced most likely by the recombinant protein overloading. Knowledge of PBs protein composition is likely to be useful to optimize downstream purification of recombinant proteins in molecular farming applications.

## Background

Plants offer an alternative to microbial fermentation and animal cultures for the production of recombinant proteins. Transgenic plants have come to be recognized as viable and efficient bioreactors for the large-scale production of recombinant proteins including pharmaceuticals and industrial enzymes [1,2]. Several approaches have been used for the improvement of yields and stability of recombinant proteins in tissue expression and subcellular targeting of recombinant proteins [3-6]. The targeting to the endoplasmic reticulum (ER) presents the advantage that ER provides an oxidizing environment, an abundance of chaperones and a low protease activity, the most important factors affecting folding, assembly and post-translational modifications [7-9]. For efficient production of foreign proteins, however, plants should still overcome two main bottlenecks including protein accumulation levels and efficient purification procedures. As in bacteria and animal protein production platforms [10], fusion strategies have been developed in plants [11]. One example is elastin-like polypeptides also called thermally-responsive synthetic biopolymers. They are composed of a repeating pentapeptide 'VPGXG' sequence [12] that accumulate in ER-derived protein bodies, when fused to a target protein and can be isolated by 'inverse transition cycling' [13,14]. Recently the use of hydrophobins (HFBI) has been reported as fusion partner for recombinant protein production in plants [15]. HFBI fusions were able to increase the accumulation of GFP in plants through the formation of novel protein bodies and purified by surfactant-based aqueous two-phase system.

Zera protein, the proline-rich domain derived from the maize seed storage protein γ-zein, is a peptide of 93 amino acids that is able to induce the formation of dense endoplasmic reticulum-derived protein bodies (PBs), when fused to target proteins. This can facilitate the recovery and purification of fused recombinant proteins by density-based separation methods [16]. The PB inducing capacity of Zera protein was demonstrated in a large panel of eukaryotes including mammalian, insect and fungi cells [17] and there are evidences that it serves also as efficient fusion partner for recombinant protein production in plants [17-19]. When a protein of interest was fused to Zera and expressed in different plant species, the fusion protein can be obtained in induced PBs in a highly packed and encapsulated manner. These vesicles could derive from general ER mechanisms able to insulate protein multimers or aggregates and segregate them away from the secretory pathway and from both the vacuolar and ER-associated degradation (ERAD) pathways [20]. This ubiquitous Zera behavior indicates the existence of intrinsic molecular properties responsible of Zera self-assembly, protein fusion polymerization and hence, PB formation. It was previously shown that the most important traits of Zera sequence are *(i) *the presence of cysteine residues, which participate in the inter-disulfide bonds between Zera sequences and *(ii) *the amphipathic feature of proline-rich repeat region which determines the efficiency of Zera-Zera self-assembly by hydrophobic interactions [19].

However, the protein composition of these Zera-induced PBs remains to be elucidated. Our aim in the present study was to characterize the final protein composition of induced protein bodies in order to understand which proteins participates in PB formation and which ones are involved in an ER-stress response or just be present by a passive trapping in Zera-DsRed polymer. *N. benthamiana *was selected as a host system and previously established protocols were adapted to isolate and characterize PBs from leaves transformed with Zera-DsRed by agroinfiltration.

## Results

### Isolation of Zera-induced PBs in *N. benthamiana *leaves

The cDNA sequence of the red fluorescent protein of *Dictyostelium discoideum *(DsRed) was fused at its N-terminus to the DNA sequence coding the signal peptide and the proline-rich domain (Zera) of γ-zein. The entire chimeric Zera-DsRed gene was inserted to the plant binary vector pCambia 2300 under the control of 35S CaMV promoter, a TEV translational enhancer and the 35S terminator. The construct was coagroinfiltrated into *N. benthamiana *leaves together with a vector containing the coding sequence of the HcPro protein, a suppressor of silencing.

**Figure 1:**
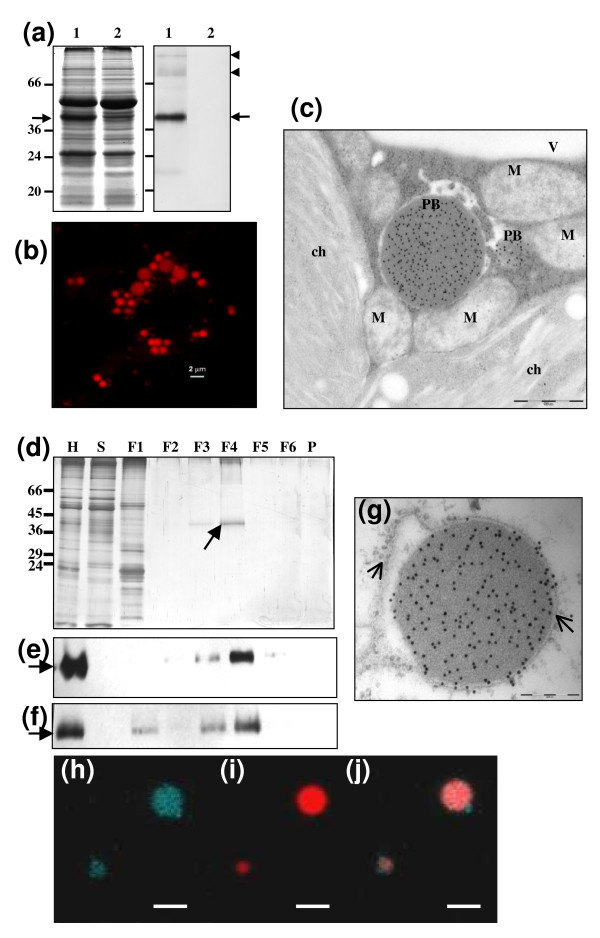
**Zera-DsRed expression in *N. benthamiana *leaves and protein bodies isolation**. (**a**) SDS-PAGE analysis and immunoblot of proteins extracted from *N. benthamiana *leaves co-infiltrated with Zera-DsRed and the silencing suppressor HcPro. Leaf tissue was collected at 7 days post infiltration (dpi). The Coomassie blue-stained gel (left panel) was loaded with 15 μg of total protein extracted from both, Zera-DsRed transformed leaf tissue (lane 1) and untransformed leaf tissue (lane 2). The gel for the immunoblot was loaded with 1 μg of of total protein extracted from Zera-DsRed transformed leaf tissue (lane 1) and untransformed leaf tissue (lane 2). Immunoblot was labelled with anti-R8 antibody (dilution 1:8000). Arrows indicate Zera-DsRed band and arrowheads in immunoblot indicates large oligomers of Zera-DsRed. (**b**) Confocal microscopy image of induced protein bodies (red) in epidermal cells transformed with Zera-DsRed and collected at 7dpi. (**c**) Immunoelectron microscopy of a thin section of leaf cell transformed with Zera-DsRed. Zera-DsRed protein inside PB was labelled with anti-R8 antibody- protein A conjugated with gold particles (10 nm). **PB**: Protein body; **ch**: chloroplast; **M**: mitochondria, scale bar: 200 nm (**d**) Protein analysis by SDS-PAGE followed by silver staining of the various fractions of the PBs isolation by density gradient. **H **is the homogenate of the leaf tissues loaded in the gradient, **S **is the supernatant after centrifugation, **F1- F6 **are the interphase fractions in the increasing densities and **P **is the pellet. Equivalent volumes were loaded in lanes **F1-F6 **and **P**, in **H **and **S **only one-third equivalent was loaded. **(e and f) **Immunoblot of the gradient fractions using anti-R8 (**e**) and anti-DsRed (**f**) antibodies. Arrows indicated Zera-DsRed protein present in the homogenate and enriched in the dense F4 fraction. (**g**) Immunoelectron microscopy of a thin section of isolated Zera-DsRed PB labeled with anti-R8 antibody-Protein A conjugated to gold particles (10 nm), arrows indicated ER membranes with ribosomes, scale bars 200 nm. (**h**-**j) **PBs isolated from leaves co-infiltrated with Zera-DsRed and ECFP-SQS1 were monitored by confocal microscopy. **(h) **Image of cyan fluorescent label of ER-membrane protein SQS1. **(i) **Fluorescent label of isolated Zera-DsRed PBs (red) and **(j) **picture shows the merged fluorescent signals and illustrated the presence of membrane protein ECFP-SQS1 surrounding isolated PBs. Scale bar: 2 μm.

Transiently transformed *N. benthamiana *leaves were collected and staining of proteins resolved on gels showed several bands, one of them being the most abundant and corresponding to Zera-DsRed (43 kDa) (Figure 1a, arrow). Zera fusions are known to oligomerize forming large polymers resistant to detergents [16] then additional bands corresponding to high molecular mass oligomers of Zera-DsRed were also observed at the top of the gel (Figure 1a, arrowhead). As expected, Zera-DsRed expression induced the formation of new organelles similar to that described for other Zera-derived fusions [17]. Under confocal microscope, Zera-DsRed protein bodies (PBs) appeared as numerous well defined highly fluorescent red spherical structures with diameters of 2-2.5 microns at 5-6 days post infiltration (dpi) (Figure 1b). Moreover, immunoelectron microscopy of transformed leaf epidermal sections labeled with a polyclonal antibody against Zera (anti-R8) detected protein bodies as electron dense structures embedded in the cytoplasm and surrounded by other organelles including mitochondria and chloroplasts (Figure 1c).

Our next aim was to investigate the protein composition of induced PBs by using proteomic approaches. PBs were isolated by density gradient procedures taking into account the well acknowledged limitations of organelle purity following density centrifugation [21]. We considered two factors to be important in optimizing the PB isolation procedure for proteome analysis: *(i) *the purity of the PB preparation and *(ii) *the intactness of the organelle. Immunocytochemical studies on thin sections of Zera-DsRed transformed leaves labelled with anti-R8 antibody indicated that Zera-DsRed PBs were derived from the ER and appeared surrounded by a typical ER membrane containing ribosomes (Figure 1g). We took advantage of the high density properties inherent to these Zera-fusion induced organelles to isolate PBs by subcellular fractionation [16]. In order to optimize the purification procedure, various density gradients based on sucrose and iodixanol (Optiprep, Axis-Shield) were checked. The different fractions obtained were analysed by protein electrophoresis and fluorescence microscopy and finally a discontinuous density gradient of iodixanol consisting of seven steps with densities ranging from 1.12 g/cm^3 ^to 1.27 g/cm^3 ^was selected to purify protein bodies for proteomic studies (see the Materials & methods section). Analysis of protein profile of all gradient-purified fractions on silver-stained SDS polyacrylamide gels (Figure 1d) and Western immunoassays with anti-R8 (Figure 1e) and anti-DsRed antibodies (Figure 1f) confirmed the presence of recombinant protein Zera-DsRed mainly in the dense F4 fraction (1.21-1.23 g/cm^3 ^interphase). The bulk of the other leaf proteins were accumulated in supernatant (S) and in the microsomal F1 fractions (Figure 1d). The adequacy of the selected PB purification procedure for isolating intact PBs was then assessed by checking whether the ER membrane surrounding the PBs is preserved after the isolation procedure. For this, we co-transformed *N. benthamiana *plants with a gene construct ECFP-SQS1 along with Zera-DsRed. The construct ECFP-SQS1 codes for the ER transmembrane domain of squalene synthase (SQS1) of *Arabidopsis thaliana *[22] fused towards N-terminus to a fluorescent protein oriented at the cytoplasmic side. The co-infiltrated leaves with ECFP-SQS and Zera-DsRed were used to isolate protein bodies and the fraction F4 of protein bodies observed under confocal microscope for the presence of the membrane envelope (Figure 1h). We observed the cyan fluorescence label of membrane protein ECFP-SQS surrounding the red-labeled protein bodies indicating the preservation, either partial or complete, of the membrane envelope during the isolation procedure of protein bodies. Thus, both the immunoelectron microscopy data described above (Figure 1g) and confocal microscopy supported the conclusion that PBs were surrounded by ER membrane.

### PBs display a complex organization and PB proteome analysis reveals an unexpected complexity

**Figure 2:**
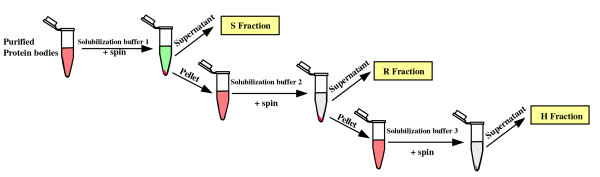
**Workflow for the sequential extraction of PB proteins**. The PB extract purified on iodixanol gradient was resuspended and incubated in a Laëmmli buffer without reductant (solubilization buffer 1) to extract soluble and membrane proteins. After centrifugation, the supernatant (S) was collected and the pellet was resuspended in a complete Laëmmli buffer (solubilization buffer 2) to extract proteins linked through disulfide bridges. The supernatant (R) was collected and the pellet was resuspended in a Laëmmli buffer and heated at 95°C for 5 min to solubilize the PB core. The supernatant was collected (H).

**Figure 3:**
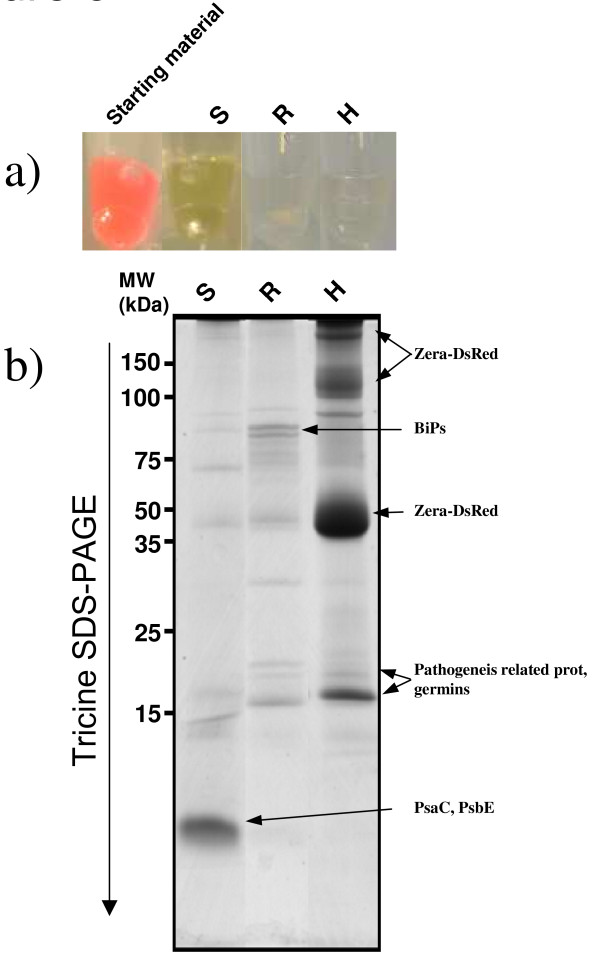
**Differential protein population of Zera-DsRed PBs**. **a) **color of the different supernatants extracted from the initial PB sample purified on Iodixanol gradient. **b) **Tricine SDS-PAGE analysis of proteins sequentially extracted from purified Zera-DsRed PBs with Laëmmli buffer in the absence of reductant (S), in the presence of 100 mM DTT at room temperature (R) and in the presence of 100 mM DTT after heating at 95°C for 5 min. In each extraction step, samples were centrifuged at 15000 g and supernatants loaded in the gel. Arrow indicates Zera-DsRed protein. c) Western immunoassay of a total protein extract of tobacco leaves (1) and PB fraction (2) using anti BiP antibodies. Lane 3 is the control lane loaded with protein extract from non transformed leaves.

**Figure 4:**
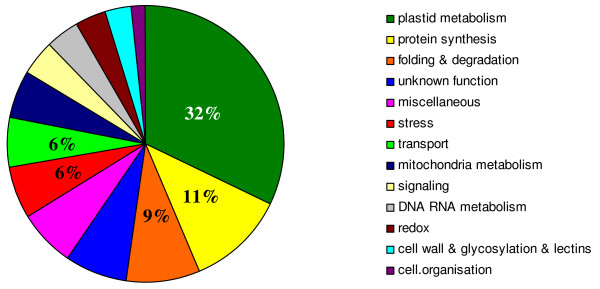
**Distribution of PB proteins in functional categories**. Percentage of the different functional categories is only indicated for the categories mentioned in the text.

In order to reduce the complexity and improve the depth of the analysis, we selected a sequential protein solubilization strategy where after incubation in a solubilization buffer, the putative pellet appearing at step n was resubmitted to solubilization at step n + 1 with a stronger solubilization buffer (Figure 2). For this purpose, the same sample was processed throughout and progressively extracted protein were collected. Namely, the initial pellet of PBs purified on iodixanol gradient was first resuspended in SB1 (a Laëmmli buffer without reductant). After incubation in this buffer, the supernatant was collected (S) and the insoluble proteins were pelleted. The pellet was then resuspended in the second solubilization buffer SB2 (a Laëmmli buffer plus reductant). Once again, the supernatant was collected (R) and the remaining insoluble proteins were pelleted and resuspended in SB3 (a Laëmmli buffer + reductant + heating at 95°C for 5 min). By this procedure, we expected SB1 would solubilize proteins outside the PB core (e.g. proteins from the ER membrane including peripheral and integral membrane proteins as well as soluble proteins present inside the PB vesicles or adsorbed on the ER membrane). SB2 would remove proteins mostly bound to the core of Zera-DsRed aggregate via intermolecular disulfide bounds or would release from the core trapped proteins and some Zera-DsRed monomers by decondensing Zera-DsRed aggregates (Zera-DsRed proteins contain several disulfide bridges essential for PB formation/aggregation). Proteins sequentially extracted from purified PBs were analyzed by SDS-PAGE. Figure 3a shows the strong enrichment of the Zera-DsRed protein in the PB extract before the sequential extractions with a pink color given by the DsRed. The greenish color of S fraction suggests the presence of plastidial proteins, whilst the colorless R fraction emphasizes the efficient extraction of these chlorophyll-bound proteins by SB1. Heating gives a colorless H fraction containing mainly Zera-DsRed protein. It should be noted that 100% of fraction S and R but only 10% of fraction H were loaded on the gel suggesting that S and R represents only a small fraction of the PB extract and shows that the latter is mainly composed of Zera-DsRed protein (Figure 3b arrow in lane H). The 3 SDS-PAGE lanes containing S, R, and H fractions (Figure 3b) were sliced into small squares (even in colorless region) and proteins were reduced, alkylated and trypsine digested. Peptides were analyzed through ESI-MS/MS and generated data were queried against the NCBInr database, Viridiplantae (release 20101018) using the Mascot search engine (see the Methods section). Additional file 1: Table S1 shows the 662 non-redundant peptides identified in the different fractions S, R, H (Additional file 1: Table S1, first sheet, column B) and the corresponding plant accession number (Additional file 1: Table S1, first sheet, column A). As the tobacco genome was not completely sequenced, the results were blasted against the *Arabidopsis *genome in order to get a better insight and remove redundancy. 84% of the identified peptides (against NCBI nr) were found to be proteotypic (meaning that the identification for these peptides is not ambiguous) and the 420 proteins identified during the first search (NCBInr, Viridiplantae) were shown to correspond to 195 different *Arabidopsis *proteins (in the *Arabidopsis *TAIR9 database). In order to highlight specific pathways or protein functions that could be altered through the production of PBs, the 195 protein accessions were classified into 13 different functional categories using a simplified version of MapMan categories [[23,24]; http://ppdb.tc.cornell.edu/] (Additional File 2: Table S2; Figure 4). Strikingly, one third of the proteins showed chloroplastic origin, among which the majority (65%) belonged to thylakoids. Roughly, another third was related to folding, stress, redox, signaling and cell wall and were probably directly connected to the overexpression and/or the secretory pathway. 11% were related to protein synthesis and represent especially ribosomal proteins. These ribosomal proteins were probably bound to the cytoplasmic side of the ER membrane and were dragged during PB purification. 1.5% of proteins related to the cytoskeleton and 4% of protein were related to DNA/RNA metabolism. 5% were related to transport protein while almost 6% had mitochondrial origin. Finally, 13% belonged to small categories (miscellaneous) or had no known functions. Nevertheless, some of these proteins seemed to be part of the secretory pathway like a cytochrome P450- related protein (AT4G37370), a strictosine synthase family (AT3G57030) or a lesion related protein (AT4G14420) in agreement with several lanes of evidence like predicted location (Additional file 2: Table S2 lane G, H) and location by mass spectrometry (lane I).

Regarding SB1 extracted fraction (S), 131 proteins were identified (Additional file 2: Table S2). One third of them were only found in this fraction and 65% of these latter were predicted to display at least one transmembrane domain (Additional file 2: Table S2). Overall, this fraction was dominated by proteins from chloroplast origin (42%) and contained 11% of proteins with function in protein and/or amino acid metabolism and 8% of proteins involved in transport. Other protein categories accounted for a minor part (less than 5% per class) (Additional file 2: Table S2).

In a second step, the resulting pellet was submitted to a complete Laëmmli buffer (SB2) containing a high concentration of reductant and 78 proteins were identified in the derived supernatant. Again, one third of these proteins were specific of the fraction, but the functional pattern was rather different. When compared to the previous fraction, both the proportion of chloroplastic proteins and proteins involved in protein and/or amino acid biosynthesis decreased by a factor of two. Conversely, proteins involved in protein folding, degradation and stress responses were concentrated by a factor of three (Additional file 2: Table S2). Finally, when the pellet was heated in Laëmmli buffer for 5 min Zera-DsRed protein was efficiently solubilized,. In addition of Zera-DsRed protein, 101 proteins were found in this fraction (H) with roughly the same global content than in the previous unheated fraction (R).

### Zera-DsRed PB's formation recruits endoplasmic reticulum proteins and traps secretory proteins

**Figure 5:**
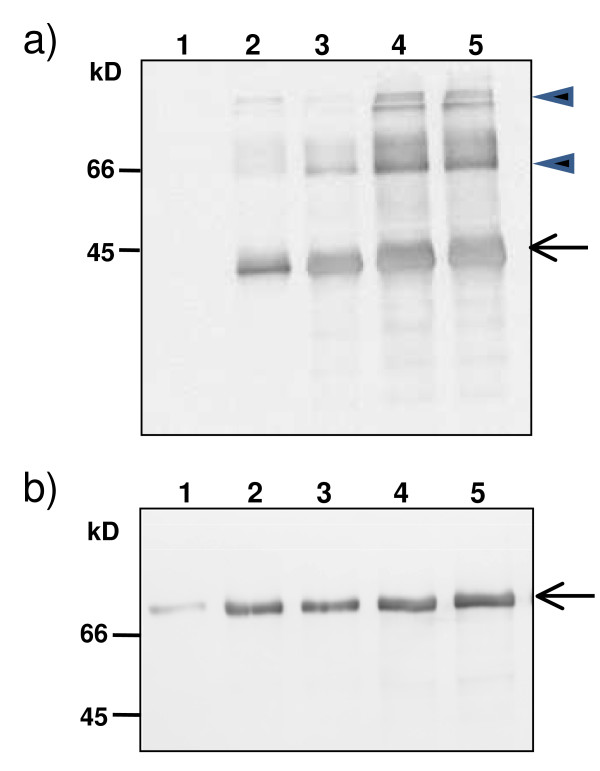
**BiP induction/stabilization during Zera-DsRed expression**. **a) **immunoblot incubated with anti-R8 antibody (dilution 1:8000). The gel was loaded with 5 μg of total proteins extracted from untransformed tobacco leaves (lane 1), Zera-DsRed transformed leaves after two days post infiltration (lane2), four days post-infiltration (lane 3) six days after post-infiltration (lane 4) and ten days post infiltration (lane 5). Arrow indicates Zera-DsRed protein, arrowheads indicated oligomers of Zera-DsRed reactive to R8 antibody. **b) **Immunoblot incubated with anti-BiP antibody (dilution 1:2000). The gel was loaded with 5 μg of total proteins extracted as in a). Arrow indicates the presence of BiP protein in protein extracts.

Besides chloroplastic proteins, 36% of the remaining proteins identified in PBs were predicted to enter the secretory pathway. In eukaryotes, secretory and transmembrane proteins are synthesized on the rough ER and translocated into the ER lumen. We then focused the study on predicted secretory proteins because they were more likely to get involved in PB formation. Secretory proteins in PBs may have been trapped during their formation or may have a more specific role towards PBs as for instance helping to fold/degrade Zera-DsRed protein or to reduce unwanted S-S bonds in the overexpressed protein. Interestingly, three functional categories cumulated half the proteins predicted to be secreted (Additional file 2: Table S2). These included proteins involved in folding, stress responses and cell wall. Among them, we found headstone folding ER proteins *i.e*. BiP and Shepperd. BiP has been shown to be highly enriched in seeds on the periphery of natural PBs [25,26] and also helping to retain and assemble prolamins into an intracisternal inclusion granule [27,28]. The presence of BiP protein in the PB preparation was confirmed using Western immunoblot (Figure 5) and showed also its induction during PB formation. Calnexin (CNX) and calreticulin (CRT) are other well-known ER resident proteins that are involved in multiple cellular processes such as protein folding and calcium homeostasis. Two isoforms of calreticulin (CRT2 and CRT3) and CNX1 were found in PB proteome. Calreticulins belong to two subfamilies in plants, one more specialized in chaperone activity (CRT1, CTR2) and the other one associated with immune responses (*e.g*. PAMP: pathogen-Associated Molecular Pattern) [29]. We cannot speculate the impact of the Zera-DsRed overexpression on the expression/activities/location of these chaperones because, through PB purification, we have a limited access to the overall secretory pathway. Furthermore, it has been shown that overproduction of recombinant proteins can disturb normal ER retention and protein sorting inducing aberrant localization of ER resident proteins such as CRT and other chaperones as BiPs in the periplasmic space [30]. Nevertheless, referring to the SDS-PAGE, BiPs are part of the more intense band (after Zera construct) and probably play important folding role during PB formation. Quantitation of PB proteome was far beyond the scope of this work. However, abundant proteins are usually identified with more than one peptide. Interestingly, most folding proteins and accessions belonging to cell wall and stress categories were usually identified with several peptides, suggesting that proteins predicted to be secreted were also fairly abundant (Additional file 2: Table S2, column G).

**Figure 6:**
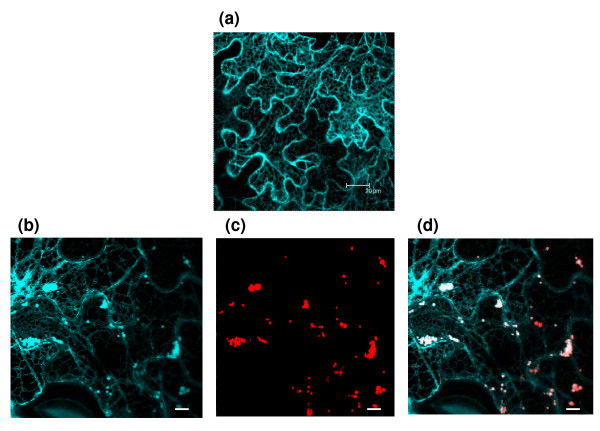
**Recruitment of ER-soluble proteins in Zera-DsRed PBs**. Confocal microscopy images of *N. benthamiana *epidermal cells **a) **transformed only with SP-ECFP-KDEL construct, scale bar 20 μm. **b**-**d) **co-transformed with Zera-DsRed and SP-ECFP-KDEL constructs. (b) ER labelled (cyan) by the expression of ECFP-KDEL, a luminal ER protein. (c) Fluorescent spots (red) corresponding to Zera-DsRed-PBs and (d) Image shows the merge of both fluorescent labels illustrating the presence of luminal-ER proteins in PBs. Scale bar: 2 μm.

In order to get additional information about the possible passive recruitment of ER resident proteins by Zera-DsRed polymers without any functional implications, we cotransformed tobacco leaves with Zera-DsRed and SP-ECFP-KDEL constructs. With the signal peptide of γ-zein (SP) and the ER retention signal KDEL the fluorescent protein ECFP will behave as a typical soluble ER-lumenal protein. The co-infiltrated tobacco leaves were observed under confocal microscope after 6 dpi. As shown in Figure 6, SP-ECFP-KDEL displayed a typical ER pattern and Zera-DsRed fluorescent spots corresponded to PBs. In addition, the merge of images evidenced the co-localization of both proteins in the protein bodies (Figure 6), reinforcing the conclusion that a significant variety of ER-soluble proteins could be recruited in Zera-DsRed-induced PBs, including some without link to PBs formation. One example of such proteins concerns the cell wall proteins, all predicted to normally enter the secretory pathway and that can be speculated to coprecipitate during PB formation, alike with the test construct SP-ECFP-KDEL. This subset includes glycosidases and glycosyl transferases (*e.g*. DGL1), that participate to the QC in the ER and cross-talk with chaperones as BiPs. DGL1 is an essential protein subunit of the oligosaccharyltransferase complex, which is responsible for the transfer in the ER of the N-linked glycan precursor onto Asn residues of candidate proteins [31]. It is relevant to point here that, when transiently expressed as Zera-xylanase in PBs of transformed tobacco, the xylanase enzyme was actually post translationally modified with N-linked high mannose type glycans [32].

### Zera-DsRed PB's formation induces expression of stress proteins in the secretory pathway

Among the three main categories rich in proteins predicted to be secreted, 3 cysteine-type proteases and one aspartyl-type have been found in PBs beside folding proteins. These proteases, that are mainly expressed in senescent leaves and seeds according to transcriptomic data [[33]; http://www.bar.utoronto.ca/], may likely not represent housekeeping enzymes. We speculate that these proteases have been probably induced in response to the Zera-DsRed overexpression. Degradation still represents a major hurdle for the production of recombinant proteins in plants and, among approaches aimed to minimize the problem, protein targeting to the cell secretory pathway has been shown to improve the stability, activity and yield of several recombinant proteins [34]. Subunits of proteasome and F-box proteins have also been found and may reflect the recruitment of proteasome at the ER surface. Indeed, it is known that secretory and membrane proteins that fail to fold in the ER are selected to ERAD and retrotranslocate to the cytosol for ubiquitin- and proteasome-mediated degradation [35].

Twelve stress proteins were found and half of them (AT2G14580, AT4G33730, AT4G11650, AT2G15220, AT3G12500, AT2G21060) are usually not present in leave tissue grown in regular conditions according to transcriptomic data [[33]; http://www.bar.utoronto.ca/]. Among stress proteins, 4 pathogenesis-related protein, 2 germin-like proteins and BGL2 were found. These proteins participate to the so-called systemic acquired resistance and their expression is usually induced by salicylic acid. Infiltration of *Agrobacteria *in tobacco leaves can potentially explain a transient induction of these proteins. However, stable transformation of Zera-DsRed protein in *Arabidopsis *shows, after several generations, the same proteins bound to PBs (data not shown). Thus, their induction is probably related to the permanent stress sensed in the ER due to the overexpression and PB formation.

## Discussion

### Proteome composition of Zera-DsRed PBs

In plant cells, Zera-DsRed over-expression induced the formation of new organelles derived from ER compartment. Indeed, Zera fusions oligomerize forming large polymers resistant to detergents named protein bodies. These structures are as big as chloroplasts and can easily be seen through a regular microscope. We optimized the purification procedure of these PBs by using a discontinuous density gradient of iodixanol and we confirmed the presence of Zera-DsRed mainly in a dense fraction (1.21-1.23 g/cm^3 ^interphase) by Western immunoassays. We also checked the intactness of PBs and specifically the presence of the surrounding ER membrane by co-transforming *N. benthamiana *plants with a gene coding for an ER transmembrane protein (ECFP-SQS1) along with Zera-DsRed. The observations under confocal microscope and the immunoelectron microscopy data confirmed the presence of the ER membrane either partial or complete, around most PBs. The bright pink color of the purified sample showed the high degree of enrichment of the construct. In a second step, to get insights into PB proteome composition and putatively also in PBs structure, a sequential extraction of PB proteins using different successive solubilization buffers was used and *de facto *increased the dynamic range of the protome analysis. The first 2 buffers used (SB1 & SB2) were not able to solubilize the PB core but solubilized soluble and membrane proteins and removed proteins bound to the core via disulfide bridges. Regarding the subset of membrane proteins, strikingly, very few ER transmembrane proteins were identified. As many proteins with predicted transmembrane domains were identified, including thylakoidal, mitochondrial and vacuolar membrane proteins, a general failure of the extraction procedure to solubilize hydrophobic proteins can be ruled out. In addition, this unexpected feature contrasts with the observation that purified PBs actually displayed a surrounding membrane able to preserve ER transmembrane proteins (Figure 1h).

Strikingly, one third of the proteins showed chloroplastic origin, among which the majority (65%) belonged to thylakoids. Previous confocal microscopic observations of isolated PB fractions did not suggest such a high level of potential chloroplastic contamination. Although this event cannot be excluded, the occurrence of attractive forces between chloroplasts and ER is also likely. For instance, chloroplasts isolated by gradient centrifugation from *Arabidopsis *plants expressing GFP in the ER lumen were shown to retain fluorescent ER fragments, indicating that the ER-chloroplasts attachments were strong enough to withstand the isolation procedure [36]. As PBs are delimited by ER membrane, this attractive force between chloroplasts and ER could be the reason for the identification of large number of proteins of chloroplastic origin in the PB proteome.

More generally, this situation posed questions about (i) the type of interactions between the ER membrane and the other organelles (plastids, mitochondria and vacuoles) (ii) the traffic of organellar proteins through the ER/secretory pathway (iii) the protein composition of the dilated ER membrane surrounding PBs. Recent data indicate that tight interactions occur between ER and different organelles. Connections between ER and chloroplast referred to as the chloroplast/ER nexus [37] or through specific extensions of the ER (ER tubules) and chloroplasts (stromules) suggest that the interacting surfaces betweeen ER and chloroplasts might serve as major conduits for bidirectional exchange of ions, lipids and metabolites between the two organelles [38-40]. Moreover, the ER and mitochondria exhibit tightly coupled dynamics and have also extensive contacts. ER tubules may play an active role in defining the position of mitochondrial division sites because mitochondrial division occurred at positions where ER tubules contacted mitochondria [41]. During PB purification, these tight interactions might explain a bandwagon effect and the presence of chloroplastic and mitochondrial proteins in PB extracts. The ripped out pieces of organellar membranes might entrap soluble proteins (e.g. Rubisco) and internal membrane (e.g. thylakoids) and could explain the occurrence of such proteins in PBs proteome. The identification of many thylakoidal proteins *vs *plastidial envelope proteins could be explained by the prevalescence of the former ones. As an alternative for chloroplast stromal proteins, it has been shown that during senescence and at times of stress, Rubisco can be mobilized in living cells via Rubisco-containing bodies using a specific autophagic pathway, which does not cause chloroplast lysis [42]. These autophagosomes have been found later in other organelles [43] suggesting that through different internal structures (tubules, stromules, autophagosomes) the plant cell can remobilize/re-allocate resources (carbon, nitrogen) to other compartments. In all cases, these organellar proteins represent a few percentage of the PBs protein abundance judged on the weak Coomassie bands seen on the SDS-PAGE (Figure 2, lane S).

The lack of ER membrane proteins is intriguing and we hypothesize that this membrane would be largely devoid of integral proteins and could constitute just a long-term storage compartment/subdomain by converting cisternal ER to spherical PB. In plants, differentiation of subdomains of the ER dedicated to protein export exists and is named "ER export sites" (ERES). This differentiation is influenced by the type of export-competent membrane cargo to be delivered to the Golgi via vesicle budding [44]. Isolated transport vesicles usually contain membrane and internal proteins that are targeted to other compartments in the cell, but they are nearly devoid of proteins that are located in the ER. Thus the budding mechanism somehow distinguishes transported from ER-resident proteins. In our case, we found ER resident proteins in PBs proteome but no ER membrane proteins suggesting that the compartment integrating PBs might be related to initial steps of vesicle budding impeded in its evolution. Transmembrane proteins are inserted in the lipid bilayer and diffuse through ER-membranes to be sorted to other compartments or retained in the ER. We can speculate that these proteins once in the ER, segregate from membrane surrounding large PBs to guarantee their diffusion and functionality.

### Response of the secretory pathway to the over-expression of Zera-DsRed

The ER is an incredibly versatile organelle that must control protein secretory processes for normal cell function and in response to perturbation of ER homeostasis.

DsRed transient overexpression and PB formation can potentially be sensed as a stress in the ER due the putative increasing need of loading and folding capacity and also to the obvious dilation of the compartment with an associated need for lipids. Two mechanisms involved in the ER integrity are the quality control (QC) and the unfolded protein response (UPR). They are mostly composed of chaperonins or other factors, proteases, signaling factors and glycosylation enzymes. Thus, analysis of a transient over-expression of Zera-DsRed leading to the formation of protein bodies can tell us how ER senses the stress and the way the plant cell uses to overcome/circumvent that stress. The ER can therefore be considered stressed when it accumulates defective proteins that have difficulty in folding [45]. BiP proteins are folding helpers and we showed an induction/stabilization of these proteins in ZeraDsRed transformed plants (Figure 5) pointing out how the plant reacts during PB formation with an overloading of misfolded proteins. We found also in PBs proteome 12 proteins classified into stress related proteins (see Additional file 2: Table S2) and according to transcriptomic data found in the literature, half of them are likely to be induced by the construct because their trancript are almost not detectable in leaves grown in normal conditions. Among these stress proteins 3 PR proteins were found and are involved in systemic acquired resistance. Proteins from other categories are also involved in or related to stress response as chaperones HSP70 (AT5G02500) or HSP90 (AT5G52640), the 3 cysteinyl proteases usually found in senescent leaves, a lesion-induced protein (AT4G14420) and a RALF 22-like protein (for Rapid Alkalinization factor) that is part of a family of polypeptide hormones that are known to regulate plant stress [46]. The overall picture suggests a stress in the secretory pathway and further investigations combining transcriptomic data and more extanded proteomic analysis (whole ER) could help to decipher the answer and adaptation of the ER facing the over-expression of proteins.

### PBs display a complex organization

**Figure 7:**
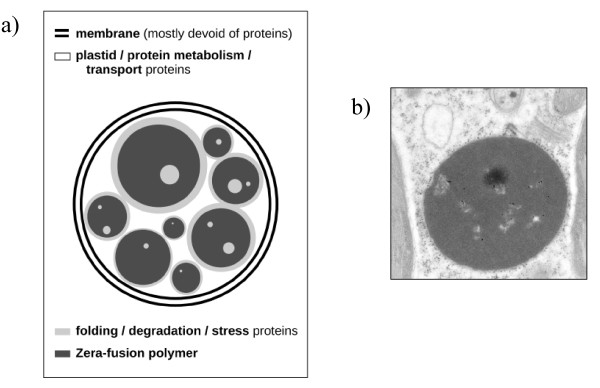
**Main features of PB's structure inferred from proteome analysis**. **a) **Both the membrane and most chloroplastic proteins as well as proteins involved in protein synthesis and transporters are directly accessible help to SDS (white regions). Most proteins involved in protein folding or degradation and in response to stress (light grey) become accessible after disruption of disulfide bonds, but part of them requires additional heating due to likely entrapment into Zera-DsRed polymers (dark grey) that resists to reductant (100 mM DTT) at room temperature. **b) **Immunoelectron microscopy of PBs showing some heterogeneity in the internal structure.

The successive extractions revealed a first set of proteins, from chloroplast and cytoplasmic origin but poor in integral ER-membrane proteins, accessible upon solubilization with a detergent. The use of a reducing agent, cleaving S-S bridges, allowed to release proteins specifically involved in folding, degradation and stress, thus highlighting the cell response to the overproduction and PB formation. These proteins contain ER retention signal or belong to the secretory pathway, further re-inforcing the *bona fide *location of these proteins. Nevertheless, a complete Laëmmli buffer was unable to solubilize Zera-DsRed unless after heating, attesting the strength of the forces involved in the assembly. Actually, recent data [19] provide strong evidence that in Zera, hydrophobic interactions, through the 8 repeats of the PPPVHL domain and disulfide bond formation by the 6 cysteine residues embedding the hydrophobic domain, are the two main driving forces for Zera self-assembly. Moreover, mutating the cysteine residues or reducing the number of PPVHL repeats often decreased significantly the formation of PBs. Owing to both the complexity of the proteome also resolved only under the most stringent condition and its function similarity to that obtained without heating, it can be speculated that the assembly of Zera-DsRed polymers would generate pockets able to entrap additional proteins (Figure 7). Thus, PBs appear to contain proteins *(i) *aggregated to Zera-DsRed polymer through "true/functional" interactions (e.g. hydrophobic, van der Waals and/or S-S bonds interactions) and/or *(ii) *trapped during Zera-DsRed polymerization and released during polymer dismantling through the action of amphiphiles and/or reducing agents.

## Conclusions

Transient overexpression of Zera-DsRed induces PB formation in tobacco leaves and PB isolation can be easily performed through iodixanol gradient centrifugation. MS/MS analysis of PBs allowed us to identified 195 proteins and shows that *(i) *the Zera construct represents *ca *85% of the total proteome in term of abundance, *(ii) *PBs contain a large number of co-purified proteins with obvious relation with (chaperones, proteases, stress proteins) or without (cell wall proteins) PB formation, *(iii) *PBs recruit ER proteins, trap secretory proteins and induce stress proteins in the secretory pathway and *(iv) *that the PB's ER surrounding membrane is mostly devoid of proteins.

## Methods

### Genetic constructs, plant transformation and growth conditions

The plant transformation vector pCZera-DsRed, containing the sequence coding for the fusion Zera-DsRed, was obtained by replacing the ECFP (Enhanced Cyan Fluorescent Protein) sequence in pUC18ZeraECFP [16] with the DsRed reporter sequence and afterwards transferring the cassette containing this Zera-DsRed sequence to the binary vector pC2300 http://www.cambia.org under the control of the enhanced 35S cauliflower mosaic virus (CaMV) promotor. Plasmid pCSPgECFP-KDEL coding for the ER resident protein SP-ECFPkdel protein was obtained as described [16].

A pUC ECFP was constructed by amplifying ECFP using pECFP-N1 (Clontech) as template adding at N terminal the restriction site SalI and AscI and BamHI at C- terminal of ECFP. SQS1 (SQualene Synthase1) coding sequence was amplified from pGFP-SQS1 [47] by appropriate primers adding the restriction sites for AscI in the Nterminal and NcoI, stop codon and BamH1 at C terminal and cloned in a pUC 18 as pUC SQS1. For making pUC ECFP-SQS1, SQS1 from pUC SQS was cloned downstream of ECFP in pUC ECFP by restriction using the enzymes AscI and BamHI. The gene construct ECFP-SQS1 was then transferred from pUC vector to pC2300 vector and named pC ECFP-SQS1.

Wild-type *Nicotiana benthamiana *plants were used for transient transformation. Plants were grown for 4-6 weeks in a greenhouse at 18-28°C, 55-65% relative humidity and a photoperiod of 16 h [48]. Individual *Agrobacterium *cultures (EHA 105 strain) transformed with binary plant vectors pCZera-DsRed, pCECFP-SQS1 and pCSPECFPKDEL were mixed with *Agrobacterium *cultures carrying the HC-Pro suppressor of silencing [49]. For transient transformation, *N. benthamiana *plants were agroinfiltrated by syringe method into the abaxial side of upper three leaves.

### Protein isolation and western immunodetection

Total soluble proteins from tissues and fractions were extracted in lysis buffer containing 0.5% SDS and 200 mM DTT for 1 h at 22°C. The various resulting extracts were centrifuged at 10000 × g for 30 min at 4°C, and proteins were separated on SDS polyacrylamide gels and detected by Coomassie blue staining or by immunoblot using the indicated antibodies. Nitrocellulose sheets were incubated with the γR8 antiserum (1:5000) antiserum for 1 h at room temperature and with anti DsRed antiserum (1:1000) over night at 4°C. The γR8 antiserum was raised in rabbits injected with the synthetic γ zein repeat domain (PPPVHLx8) coupled to the Keyhole limpet haemocyanin (KLH) protein used as a carrier [16]. The antibodies anti DsRed (anti RFP) used in this study was commercial (Abcam). Immunoreactive bands were detected by chemiluminiscence (ECL Western Blotting System; Amersham).

### Subcellular fractionation

1 g of agroinfiltrated tobacco leaf tissues were ground in a mortar at 0°C in 21 ml of an isotonic homogenization buffer (HB) containing Tris-HCl 10 mM pH 8, 0.25 M sucrose and protease inhibitors (Plant protease inhibitors, Sigma,1:250 dilution). The homogenate was filtered through two layers of Miracloth (22-24 μm, Calbiochem) to remove tissue debris and centrifuged at 100 × g for 5 min at 4°C. Resulting clarified homogenates were loaded onto multi-step Iodixanol (Optiprep, Sigma) density based gradients (steps: 1.11, 1.17, 1.19, 1.21, 1.23 and 1.25 g/cm3). For step gradients preparation, a working solution of 50% w/v (Y50) of iodixanol was made by mixing five volumes of Optiprep in one volume of 250 mM sucrose in HB buffer. Iodixanol steps were prepared from diluting of Y50 solution in HB buffer. Polyallomer centrifuge tubes of 12 ml were filled with 1.5 ml of each iodixanol step and 3 ml of clarified homogenate in the top of tube. The gradients were centrifuged at 4°C for 2 h at 80000 × g in a Beckman SW40 Ti rotor. Equivalent aliquots of supernatant, interphase fractions and pellet were analyzed by SDS-PAGE followed by protein staining in coomassie blue or immunoblot using specific antibodies. For proteome analysis, the PBs sedimented in the interphase of 1.21-1.23 g/cm^3 ^were collected by puncturing the tubes at the corresponding interphase. The yield of Zera-DsRed recovered in these interfaces was 60% of total Zera-DsRed expressed in leaf tissues.

### Immunocytochemistry and imaging

#### Confocal microscopy

PB fractions were directly observed under an FV1000 Olympus confocal microscope. Red fluorescent images were collected after 543 nm excitation using a 550-600 nm emission window. Cyan fluorescent images were collected at 458 nm excitation and an emission window of 470-530 nm.

#### Electron microscopy

For immunocytochemistry, PBs were pelleted and fixed with 1% glutaraldehyde and 2.5% paraformaldehyde in 20 mM phosphate buffer, pH 7.4, for one hour at room temperature. After washing, dehydratation and embedding in Lowicryl K4M resin, immunochemistry was performed essentially as described using antiR8 antibody and protein A-colloidal gold (15 nm) [50]. Sections were examined under an electron microscope (Phillips EM301, Eindhoven, The Netherlands). In all cases non-immune serum was used as control.

### Protein separation and identification by mass spectrometry

Proteins (5 mg/ml) were solubilized with different extraction buffers: Laëmmli buffer without reductant, complete Laëmmli buffer (including 100 mM DTT) at room temperature, and complete Laëmmli buffer with heating for 5 min at 95°C. The resulting supernatants were separated on a 12% SDS-PAGE with a Tricine buffer. Proteins were stained with Coomassie brillant blue and protein bands were picked throughout the lanes. After trypsin digestion, peptides were resolved using a linear gradient (3% to 80% acetonitrile in 0.1% formic acid; 300 nL/min) and an HPLC Chip-Cube system (Agilent Technologies) coupled to an high capacity ion trap mass spectrometer (Bruker Daltonik). Peak lists were generated with the Data Analysis software (BrukerDaltonik) and the NCBInr (release 20101018) database was queried using the Mascot search engine (v. 2.2.04) and the following parameters: trypsin as enzyme, 1 missed cleavage allowed, carbamidomethylation of cysteine as fixed modification, oxidation of methionine as variable modification and 0.6 Da mass accuracy in both MS and MS/MS. Accessions were identified at p < 0.01 with a FDR < 3%. The corresponding Mascot score that fits to the above requirement was 53. For more detailed analysis, a semi-automatic extraction of data followed by a systematic manual verification was also done using a combination of stringent criteria to validate peptides: at least 5 y, b, y++ or b++ ions, at least 4 consecutive ions, more than 2 ions in the top 10 of the more intense ions in the fragmentation spectra, an e value lower than 2, a Mascot score above 20, a score difference between the first match to the query and the second one over 10 excepted for I/L substitution and no unexpected missed cleavage.

## Authors' contributions

MJ and MT participated in experimental design of protein bodies recovery and performed plant transformations and organelle isolation from tobacco leaves. MJ also performed protein analysis and confocal microscopy. VR generated mass spectrometry data and MT participated to bioinformatics data treatment. JBP and MDL designed the approach. JBP performed protein fractionation, analysed proteomic data and participated in writing of the manuscript. MR, PC and MDL supervised the work and wrote the manuscript. All authors read and approved the final manuscript.

## Supplementary Material

Additional file 1 **Directory of proteins in PBs induced in *N. benthamiana *by over-expression of Zera-DsRed**. Sheet 1: identified peptides and proteins. Sheet 2: identification parameters according to the Method Section. Click here for file

Additional file 2 **Features of proteins**. Including predicted features (protein function, number of TMD, sorting) and experimental ones (unicity of peptide/accession, fraction(s) where observed). Click here for file
