# Supplementary figures and images for: Proteomic characterisation of endoplasmic reticulum-derived protein bodies in tobacco leaves
Source: BMC Plant Biol. 2012 Mar 16;12:36. doi: 10.1186/1471-2229-12-36 (PMC3342223; doi:10.1186/1471-2229-12-36)

# Figure 2

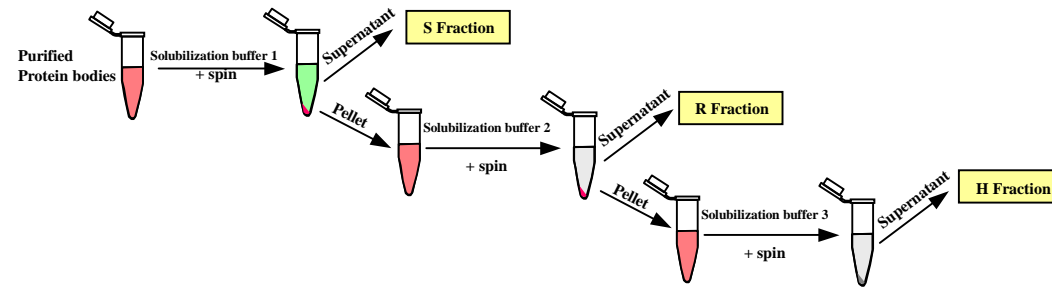

Supplement: Additional file 1 — Directory of proteins in PBs induced in N. benthamiana by over-expression of Zera-DsRed. Sheet 1: identified peptides and proteins. Sheet 2: identification parameters according to the Method Section. [file 1471-2229-12-36-S1.PDF]
